# Spatial chemical conservation of hot spot interactions in protein-protein complexes

**DOI:** 10.1186/1741-7007-5-43

**Published:** 2007-10-09

**Authors:** Alexandra Shulman-Peleg, Maxim Shatsky, Ruth Nussinov, Haim J Wolfson

**Affiliations:** 1 School of Computer Science, Raymond and Beverly Sackler Faculty of Exact Sciences Tel Aviv University, Tel Aviv 69978, Israel; 2 Physical Biosciences Division, Berkeley National Lab, California, USA; 3 Basic Research Program, SAIC-Frederick, Inc. Center for Cancer Research Nanobiology Program, NCI, Frederick, MD 21702, USA; 4 Department of Human Genetics and Molecular Medicine Sackler Faculty of Medicine, Tel Aviv University, Tel Aviv 69978, Israel

## Abstract

**Background:**

Conservation of the spatial binding organizations at the level of physico-chemical interactions is important for the formation and stability of protein-protein complexes as well as protein and drug design. Due to the lack of computational tools for recognition of spatial patterns of interactions shared by a set of protein-protein complexes, the conservation of such interactions has not been addressed previously.

**Results:**

We performed extensive spatial comparisons of physico-chemical interactions common to different types of protein-protein complexes. We observed that 80% of these interactions correspond to known hot spots. Moreover, we show that spatially conserved interactions allow prediction of hot spots with a success rate higher than obtained by methods based on sequence or backbone similarity. Detection of spatially conserved interaction patterns was performed by our novel MAPPIS algorithm. MAPPIS performs multiple alignments of the physico-chemical interactions and the binding properties in three dimensional space. It is independent of the overall similarity in the protein sequences, folds or amino acid identities. We present examples of interactions shared between complexes of colicins with immunity proteins, serine proteases with inhibitors and T-cell receptors with superantigens. We unravel previously overlooked similarities, such as the interactions shared by the structurally different RNase-inhibitor families.

**Conclusion:**

The key contribution of MAPPIS is in discovering the 3D patterns of physico-chemical interactions. The detected patterns describe the conserved binding organizations that involve energetically important hot spot residues and are crucial for the protein-protein associations.

## Background

Protein-protein interfaces (PPIs) are defined as regions of interaction between two non-covalently linked protein molecules. As binding is closely related to function, analysis of the properties of PPIs have long been a problem of major interest [1-7]. The pioneering work of Clackson and Wells has shown that only a small and complementary set of cooperative contact residues, termed "hot spots" maintains the binding affinity [8]. Hot spots are identified by alanine scanning experiments. They are defined as residues whose mutation to alanine leads to a significant drop in the binding free energy [9,10]. Several works have studied the nature and organization of hot spots [11-13] as well as their computational prediction [14-19]. Using the double mutant cycle, Schreiber and Fersht have shown the cooperativity of residues and interactions across the interface [20]. Furthermore, it was shown that PPIs are built in a modular fashion [21] and there is a cooperativity between the hot regions [22] and the conserved residues [23,24].

A key underlying concept in many studies postulates that functionally important properties are conserved throughout evolution [13,25] and can be recognized by the comparison of a set of protein sequences [26-29] or structures [30-32]. Structural classification of protein-protein interfaces by their *C*_*α *_patterns [33,34] has led to an insight into interface organizations [35] and preferred residues conformations [36]. However, backbone atoms do not fully capture the physico-chemical nature of the interfaces and chemical interactions are known to be created by atoms of side chains with different residue identities. Current methods that do compare physico-chemical properties align single binding sites (i.e. one side of the interface) and do not consider the interacting partner [37-40]. Recently, we have presented a method that aligns a pair of PPIs by simultaneously considering the two pairs of complementary binding sites [41,42]. However, a combination of high scoring pairwise patterns does not necessarily provide a high scoring pattern common to a set of PPIs [43]. Several studies considered the chemical interactions formed across the interface [44-46] and used them for classification [47,48] and complex prediction [49]. However, the spatial conservation of these interactions was not systematically addressed, mostly due to the lack of computational tools for recognition of spatial patterns of interactions shared by a set of PPIs.

Here, we present the first extensive study of the spatial conservation of physico-chemical interactions shared within families of PPIs formed by functionally similar proteins. This study was performed with our novel method, MAPPIS (Multiple Alignment of PPIS). The method is based on physico-chemical interactions formed across the interface between groups of atoms, which may derive from amino acids with different identities and backbone locations [50]. The uniqueness of MAPPIS lies in its ability to detect spatially conserved patterns of interactions even when there is no sequence and fold homology between the corresponding proteins. By applying MAPPIS to different families of PPIs, we observed that (i) most of the conserved physico-chemical interactions are contributed by the hot spot residues, and (ii) consequently, MAPPIS predicts hot spots with a high success rate, indicating the functional importance of the conserved chemical interactions. Using MAPPIS, we further provide specific biological examples that reveal previously overlooked similarities between structurally different though functionally related complexes.

## Results and discussion

We assess the significance of spatially conserved patterns of interactions. First, we describe the physico-chemical patterns we look for and the concept behind MAPPIS. Next, we present an extensive analysis of the families of PPIs that were previously studied by experimental alanine scanning, and show that spatially conserved interactions can predict hot spots. Finally, we provide the details of the specific patterns of interactions shared within these families.

### Recognition of shared interactions

**Figure 1:**
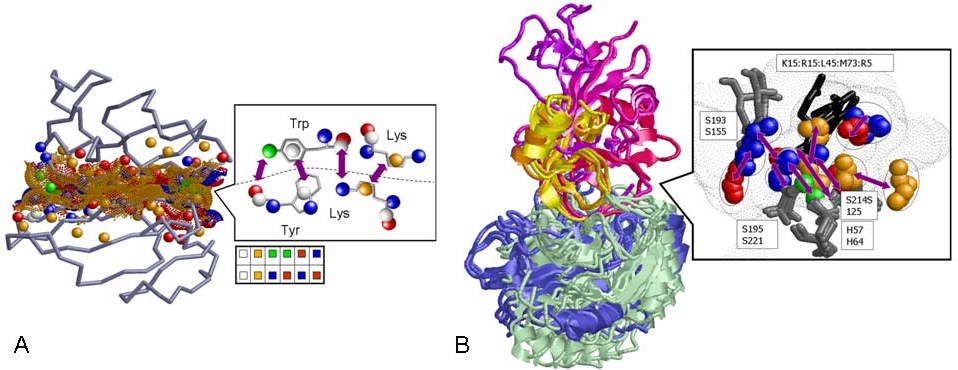
**Shared interactions**. (**A**) The left figure shows a PPI represented by the solvent accessible surfaces (small dots [51]) and the pseudocenters (balls). Only surface exposed pseudocenters are considered. Hydrogen bond donors are blue, acceptors – red, donors/acceptors – green, and aromatic – white. The right figure illustrates the definition of pseudocenters and the bar at the bottom illustrates the complementarity of the pseudocenter properties. (**B**) Alignment of 6 PPIs of serine proteases with inhibitors. The trypsins (1cbwHG, 1tawA, 1ca0HG) are gray and the subtilisins (1cseE, 2sicE, 1oyvB) are blue. The corresponding inhibitors (1cbwI, 1tawI, 1ca0I, 1cseI, 2sicI, 1oyvI) are colored ranging from yellow to purple respectively. The right figure presents the 9 spatially conserved interactions (purple arrows). The catalytic residues of the serine proteases (gray sticks) were recognized to form 5 similar interactions (3 hydrogen bonds, 1 hydrophobic aliphatic and 1 aromatic) with the corresponding hot spots of the inhibitors K15(1cbw), R15(1taw,1ca0), K45(1cse), M73(2sic) and R5(1oyv). These residues, which have different amino acid identities and backbone locations are represented as black sticks.

A PPI is defined by a pair of interacting binding sites. The area of each binding site is determined by the solvent accessible surface points [51] that are located less than 4Å from the surface of the binding partner. Following the definition of Schmitt et al [39], each amino acid in a protein is represented by points in 3D space termed pseudocenters. Each pseudocenter represents a group of atoms according to the interactions in which it may participate: hydrogen-bond donor, hydrogen-bond acceptor, mixed donor/acceptor, hydrophobic aliphatic and aromatic (*π*) contacts. Some of the atoms of a pseudocenter may be buried and some may be exposed. We considered all the pseudocenters with at least one surface exposed atom. These were assigned the following attributes: (i) charge; (ii) normal vectors that denote the surface direction and ring plane orientation (for aromatic rings); (iii) surface patch size and curvature (estimated by the solid angle shape function [52]); Figure 1A presents examples of a representation of amino acids by pseudocenters. For example, the side chain of Lys is represented by a donor, located at the nitrogen atom, and a hydrophobic aliphatic pseudocenter, located at the center of mass of its four carbons [39].

An interaction is defined by a pair of close enough pseudocenters, one from each side of the interface, possessing complementary physico-chemical properties. Specifically, hydrogen bond donors are complementary to acceptors, while hydrophobic aliphatic and aromatic centers can interact with similar ones. Pseudocenters with the mixed donor/acceptor property, such as the nitrogen atoms of His, can interact with both donors and acceptors. The interaction distance thresholds are 3.9Å [53] for hydrogen bonds and 8Å for the rest (according to the maximal possible distance between pseudocenters that represent groups of atoms). As the exact computational definition of real interactions is not straightforward [53], we practically overcame this problem by considering all possible interactions at the early stages and selecting only those that are conserved in all the complexes.

We compared the spatial arrangements of the following three interaction types: hydrogen bonds, hydrophobic aliphatic and aromatic (*π*) contacts. Two interactions are considered similar if they are created by similar pseudocenters that are superimposed to nearby spatial locations (e.g. ≤ 3Å). The similarity of pseudocenters was measured by a scoring function that compares properties like spatial proximity (after the superimposition), charge, surface curvature as well as aromatic ring plane orientation. The similarity of interactions from two different PPIs is scored according to the similarity of the corresponding pseudocenters and the complementarity of their properties. Specifically, we measured the complementarity in terms of the pseudocenter proximity, charge complementarity, surface fit as well as aromatic ring orientations (favoring perpendicular and parallel *π *stacking). Given a set of PPIs, MAPPIS finds a set of transformations that superimpose them in 3D space in a way that maximizes the spatial and chemical similarity of their interactions and pseudocenters (see Methods).

To illustrate the concept behind MAPPIS we aligned six PPIs of serine proteases with inhibitors. These are formed between serine proteases of two structural folds (trypsin and subtilisin) with inhibitors that have different structural classifications [54] and less than 4% sequence identity. Figure 1B presents a pattern of nine common interactions recognized by MAPPIS (six hydrogen bonds, two hydrophobic aliphatic and one aromatic). The correct alignment of the catalytic residues of the serine proteases indicates the accuracy of the MAPPIS solution. Studying the PPIs of trypsins, Scheidig et al [55] have stressed the importance of the interactions formed with the hot spots Lys15 and Arg15 of the trypsin inhibitors (1cbw, 1taw, 1ca0). Our results are consistent with this observation. Moreover, MAPPIS found that the PPIs of subtilisins exhibit five spatially similar interactions formed with the residues Leu45 (1cse), Met73 (2sic) and Arg5 (1oyv) of the corresponding subtilisin inhibitors. In particular, as can be seen these interactions are formed by amino acids with different identities and backbone locations. However, these amino acids have similar physico-chemical properties (pseudocenters) that form similar spatially conserved interactions. Hence, residue-based methods would not have detected these conserved (hot spot) interactions.

### Hot spot prediction

Here, we perform an extensive analysis of the available structural data and show that recognition of spatially conserved interactions can predict hot spots. We have retrieved all complexes with significant numbers of alanine mutations deposited in the AseDB database [10] and analyzed by Kortemme et al. [14].

For each such complex, we retrieved all the complexes created by molecules with the same molecule name in the PDB [56] and the same family id in SCOP [54]. As similarity of the overall sequences and structures does not necessarily implies the similarity in the binding patterns and vice versa, we did not remove sequence homologues and retained SCOP family members only if they shared more than three interactions with the constructed PPI family. Following this procedure we obtained a dataset of 12 PPI families, each with an average of six members (see Table 1).

**Table 1:** The dataset of PPI families with available alanine scanning data [10,14]. The complexes tested by experimental alanine scanning, are detailed in columns 1–3. Column 4 presents the number of PPI family members created by molecules with the same functional description by PDB [56] and SCOP [54]. In the case of multiple structures of exactly the same complexes, we have arbitrarily chosen half of the structures. Crystal structures with resolution lower than 2.5Å were ignored. Column 5 details the PDB codes and the chain identifiers of the proteins in PPIs. Column 6 provides the percentage of sequence identity between the complexes in each family, measured by structure based sequence alignment method Staccato [63])

Mutated PDB: Chain	Name	Partner	Family size	Family data set details (PDB Chain 1: Chain 2)	Seq. Id. (%)
1a4y:A	RNase inhibitor	Angio genin	4	1a4y A:B, 1z7x Z:Y, 1dfj I:E, 2bex A:C	48
1brs:A	Barnase	Barstar	6	1brs A:D, 1b2s A:D, 1b27 A:D, 1x1u A:D, 1x1w A:D, 1b2u A:D	94
1brs:D	Barstar	Barnase	6	1brs A:D, 1b2s A:D, 1b27 A:D, 1x1u A:D, 1x1w A:D, 1b2u A:D	94
1cbw:I	BPTI	Trypsin	7	1cbw I:HG, 1taw B:A, 1ca0 I:HG, 1ejm B:A, 3tgk I:E,1fak I:H, 1p2k I:A, 1f7z I:A	24
1gc1:C	CD4	gp120	6	1gc1 C:G, 1g9n C:G, 1rzk C:G, 1rzj C:G, 1g9m C:G, 1yym M:G	54
1bxi:A	Im9	E9 DNase	6	1bxi A:B, 1emv A:B, 1fr2 A:B, 1znv A:B, 1mz8 A:B, 1ujz A:B	56
1dan:L	Factor VII	Tissue Factor	6	1dan LH:TU, 1fak LH:TU, 2aer LH:T, 1wun LH:T, 1wtg LH:T, 1wqv L:T, 2a2q L:T	49
1jck:A	TCR Vb	SEC3	6	1jck A:B, 2aq1 A:B, 2aq2 A:B, 2aq3 A:B	13
1jck:B	SEC3	TCR Vb	6	1jck A:B, 2aq1 A:B, 2aq2 A:B, 2aq3 A:B	13
1vfb:C	HEL	D.1.3	6	1vfb C:AB, 1a2y C:AB, 1fdl Y:LH, 1g7h C:AB, 1g7i C:AB, 1kip C:AB	45
3hfm:Y	HEL	HYHEL	8	3hfm Y:LH, 1ua6 Y:LH, 1j1o Y:LH, 1j1p Y:LH, 1uac Y:LH, 1ic7 Y:LH, 1c08 C:AB, 1nby C:AB	57
3hhr:A	hGH	hGHbp	4	3hhr A:B, 1a22 A:B, 1axi A:B, 1hwg A:B	89

We have observed that in these families, on average 80% of the shared interactions with similar spatial physico-chemical organization are created by the hot spot residues (following Kortemme et al. hot spots are defined as residues with ΔΔ*G *≥ 1 *kcal/mol *[14]). Moreover, we show that these conserved interactions can be used to predict hot spots with a mean success rate of 0.75, calculated as the average of the true positive rate (specificity) and the true negative rate (sensitivity) of the hot spot predictions. The specificity is defined by *TN/*(*TN *+ *FP*), and the sensitivity is *TP/*(*TP *+ *FN*), where *TP *and *FP *are the numbers of true and false positives and *TN *and *FN *are the number of true and false negatives respectively. In addition, we constructed (ROC) curves, which plot the sensitivity as a function of the true negative rate (1-specificity), while varying the prediction threshold. The area under this curve indicates the performance gain over a random predictor (with an area of 0.5).

Remarkably, the average ROC area of MAPPIS is 0.77 and it is thus considered to be a good hot spot predictor. Table 2 presents a comparison of MAPPIS with two state-of-the art computational methods: Consurf [27], which calculates the evolutionary conservation within a protein family, and Robetta [14,15], which explicitly calculates the expected change in the binding free energy upon mutation to alanine. The performance of MAPPIS was significantly better than Consurf, which had a ROC area of 0.48. When compared to Robetta both methods had almost the same specificity (0.86 for MAPPIS and 0.85 for Robetta). The sensitivity of MAPPIS and Robetta were also quite similar, with only a slight difference (0.66 for MAPPIS versus Robetta's 0.64). These results show that MAPPIS captures the energetics of the protein-protein interactions and can predict hot spots with a high success rate. As computational alanine scanning methods, like Robetta [15], consider single structures, MAPPIS can not replace them. Rather, it complements them by showing the important role of the hot spots in the atomic interactions and in explaining their cooperativity. Moreover, it reveals the conserved chemical binding organizations, which are formed by the atomic interactions and can not be detected at the residue level.

**Table 2:** Prediction of hot spots with MAPPIS, Consurf [27] and Robetta [14,15]. Columns 1–4 are as in Table 1. The ROC curves were constructed by varying the MAPPIS threshold of the interaction score and the Consurf conservation grade. The sensitivity and the specificity were calculated with a MAPPIS score of 2 and Consurf score of 6, which gave the best performance. As no threshold could be varied for Robetta, we could not construct its ROC curves. The calculations were restricted to the PPI regions of the representatives as considered by MAPPIS. The last columns presents the number of mutations and hotspots retrieved from the AseDB database [10].

PDB: Chain	Name	Partner	**MAPPIS**				**Consurf**			**Robetta**		# Mutations
			# PPIs	ROC area	Spec.	Sens.	ROC area	Spec.	Sens.	Spec.	Sens.	

1a4y:A	RNase inhibitor	Angiogenin	4	0.74	0.78	0.75	0.18	0.78	0	0.75	0.8	14
1brs:A	Barnase	Barstar	6	0.75	1	0.5	n/a	n/a	n/a	0.5	0.67	8
1brs:D	Barstar	Barnase	6	1	1	1	n/a	n/a	n/a	1	1	6
1cbw I	BPTI	Trypsin	7	1	0.88	1	0.75	0.62	1	0.75	1	9
1gc1:C	CD4	gp120	6	0.74	0.86	0.67	0.58	0.62	0.67	0.81	0.33	49
1bxi:A	Im9	E9 DNase	6	0.72	1	0.44	0.72	0.88	0.56	0.88	0.44	28
1dan:L	Factor VII	Tissue Factor	6	1	0.73	1	0.23	0.64	0	0.73	1	107
1jck:A	TCR Vb	SEC3	6	0.65	0.88	0.4	0.41	0.75	0	1	0.5	24
1jck:B	SEC3	TCR Vb	6	0.75	1	0.5	0.31	0.5	0.12	1	0.62	10
1vfb:C	HEL	D.1.3	7	0.51	0.62	0.5	0.27	0.62	0.25	1	0	12
3hfm:Y	HEL	HYHEL	8	0.62	0.6	0.67	0.78	0.7	0.67	0.8	1	13
3hhr:A	hGH	hGHbp	4	0.74	1	0.52	0.59	0.44	0.68	0.94	0.32	161

			Mean MAPPIS				Mean Consurf			Mean Robetta		Total

			6	0.77	0.86	0.66	0.48	0.66	0.4	0.85	0.64	440 (116)

We compared the predictive power of MAPPIS with our previously developed multiple alignment methods. The first method, MultiProt [57] performs multiple structural alignment of the protein backbones represented by the *C*_*α *_atoms. Here, it was applied to simultaneously align the overall structures of both proteins in a complex. The specificity of its hot spots predictions was low (0.29) and due to the large number of false positive solutions it is less suitable for this purpose. The second method, MultiBind [58] is based on recognition of similar physico-chemical properties of the protein binding sites without any consideration of the binding partners. As most of the conserved interactions recognized by MAPPIS are created by regions with similar physico-chemical properties, the predictions made by MAPPIS are a subset of the predictions of MultiBind. However, as it ignores the binding partners and the interactions created across the interfaces it has a high false positive rate, its specificity is 0.44 and its area under the ROC curve is 0.58 (see table in Additional file 2). In addition, as MAPPIS utilizes the information of interactions, its running times are 10-fold faster than those of MultiBind and its average running time on a typical family of 6–7 PPIs is 3–4 minutes (on a standard PC, 2.60 GHz CPU, 2 GB RAM).

### PPIs of ribonucleases with inhibitors

Ribonucleases (RNases), which catalyze RNA degradation, are lethal to the cell when expressed without their specific RNase inhibitor (RI). The affinity of RI for RNases is one of the highest among known protein-protein complexes (e.g. 1 fM for RI-Angiogenin [59]). Below we analyze the different types of RNase-inhibitor complexes and present the interactions shared within each family as well as the interactions conserved between the PPIs formed by proteins with different overall sequences and folds.

#### Barnase-Barstar

Barnase is a bacterial protein with a RNase activity and barstar is its specific inhibitor. We aligned six PPIs of barnase-barstar (PDB:chain1:chain2 – 1brsAD, 1b2sAD, 1b27AD, 1x1uAD, 1x1wAD, 1b2uAD. See figure 1A in Additional file 1). These PPIs were recognized to share 17 interactions, which are conserved among the average of 25 interface interactions. Thirteen of them are interactions created by known hot spots in at least one PPI chain and six of them are created by pairs of interacting hot spots. These are created by Asp39 of barstar interacting with Arg-83 and Arg-87 of barnase as well as barstar Glu76 and Asp36 which interact with barnase Arg59 and His102 respectively. The importance of these interactions was experimentally validated by the double mutant studies of Schreiber and Fersht [20] who have measured that their coupling energies range from 5–7 kcal/mol.

#### RNase A-like with leucine-rich repeat inhibitors

Another type of Ribonuclease-inhibitor complex is formed by RNase A-like ribonucleases [54] with leucine-rich repeat inhibitors. We applied MAPPIS to compare the PPIs of four complexes (see figure 1B in Additional file 1): (1) RI with Angiogenin (1a4yAB); (2) RI with human eosinopil derived neurotoxin (2bexAC); (3) RI complexed with RNase I (1z7xZY) and (4) RI with RNase A (1dfjIE); MAPPIS recognized 7 interactions that are spatially and physico-chemically conserved in all the complexes (see table 2 in Additional file 2). The conserved interactions recognized by MAPPIS are formed by the known hot spots Tyr-434 and Asp-435 of Angiogenin with ΔΔ*G *of 3.3 and 3.5 *kcal/mol *respectively. Additional interactions are the *π *contacts between the rings of Angiogenin Tyr-437 with His-114 of RI, which in spite of a ΔΔ*G *of 0.8 *kcal/mol *are conserved in all the complexes. Some interactions are formed between groups of atoms and are independent of the amino acid identities. For example, the hydrogen bond between a side-chain of Tyr-434 (donor/acceptor) and the backbone *O *atom of Pro-38 (acceptor) in RI-Angiogenin complex (1a4y) is similar to a hydrogen bond formed in the RI-neurotoxin complex (2bex) by the side-chain of Tyr-434 with the backbone of Arg-36. Interestingly, the side chains of these RI residues Pro-38 in 1a4y and Arg-36 in 2bex form similar hydrophobic interactions with Val-432 in RNases, which although not experimentally tested, is predicted by Robetta [14] to be a hot spot.

#### Ribonucleases with inhibitors: different folds similar functions

**Figure 2:**
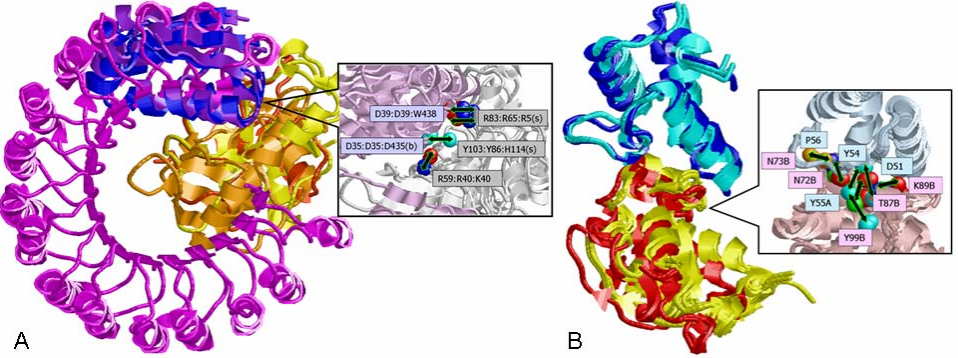
**Alignment examples**. (**A**) Alignment of 3 RNase-inhibitor PPIs. The Angiogenin is yellow (1a4yB) and the Rnase Sa (1ay7A) and barnase (1brsA) are dark and light orange. The Leucine-rich RI (1a4yA) is in magenta while the barstars (1ay7B,1brsD) are blue and purple. The rightmost figure shows the conserved interactions with the identities of the amino acids that create them in the corresponding complexes (1a4y:1ay7:1brs). The pseudocenters are represented as in Figure 1A, except the aromatic properties which are cyan. The RIs are light purple and the RNases are monochrome. (**B**) Alignment of 6 PPIs of E9-Im9 (1bxiAB, 1emvAB, 1fr2AB) and E7-Im7(1mz8AB, 1ujzAB, 1fr2AB). The E9 are cyan and the E7 are blue. The Im9 and Im7 are yellow and red respectively. The right figure details the 7 conserved interactions labeled by the amino acids of 1bxiAB.

Both of the above examples are RNase-inhibitor families that perform similar functions, but their sequences and structures are totally different. MAPPIS enables the recognition of previously overlooked spatial patterns of interactions shared by their PPIs. Specifically, we applied MAPPIS to compare between the three most distinct complexes (less than 4% sequence identity): (i) Barnase-barstar (1brsAD); (ii) RI with Angiogenin (1a4yAB) and (iii) Barstar with RNase Sa (1ay7AB). Figure 2A as well as Table 3 present the results of the MAPPIS alignment. We have recognized four interactions that are formed by known hot spots in all types of complexes [20,59].

**Table 3:** The interactions shared by PPIs of structurally different Ribonucleases with inhibitors. Each pair of rows details the interacting pseudocenters of two PPI chains. Each three columns present the details of a specific PPI: (i) chain identifier and residue number; (ii) residue type; (iii) pseudocenter type, which can be donor (DON), acceptor (ACC), mixed donor/acceptor (DAC), hydrophobic aliphatic (ALI) or aromatic (PI). The last column presents the origin of the feature: backbone(b) or side-chain(s) if it is the same for all the matched pseudocenters.

1brs:Barstar(D)-Barnase(A)			1ay7:Barstar(B)-RNase Sa(A)			1a4y:RI(A)-Angiogenin(B)			
Chain. R. Num	R. Type	Psc. Type	Chain R. Num	R. Type	Psc. Type	Chain R. Num	R. Type	Psc. Type	Type

D.35	Asp	PI:C	B.35	Asp	PI:C	A.435	Asp	PI:C	b
A.103	Tyr	PI:	A.86	Tyr	PI	B.114	His	PI	s
D.35	Asp	ACC	B.35	Asp	ACC	A.435	Asp	ACC	s
A.59	Arg	DON	A.40	Arg	DON	B.40	Lys	DON	
D.39	Asp	ACC	B.39	Asp	ACC	A.438	Trp	ACC	
A.83	Arg	DON	A.65	Arg	DON	B.5	Arg	DON	s
D.39	Asp	ACC	B.39	Asp	ACC	A.438	Trp	ACC	
A.83	Arg	DON	A.32	Gln	DON	B.5	Arg	DON	s

Specifically, we recognized two similar hydrogen bonds formed by the hot spots Asp-39 and Arg-83 of barnase-barstar and the Trp-438 and Arg-5 of RI-Angiogenin. We recognized that a hydrogen bond formed by the hot spots Asp-35 and Arg-59 in the barnase-barstar complex is similar to the hydrogen bond formed by the hot spots Asp-435 and Lys-40 in the RI-Angiogenin complex. Separately, for each type of complex the importance of these interactions has already been reported [20,59]. However, as they are created by amino acids with different identities and backbone locations, their similarity have never been detected before.

### PPIs of colicin DNases with immunity proteins

The E colicin DNases are bacterial toxins that kill target microbial cells through random degradation of chromosomal DNA. Their catalytic activity is neutralized by the respective immunity proteins (Im) [60]. We applied MAPPIS to analyze and classify the 5 types of available complexes: (i) colicin E3 DNase with Im3; (ii) E5-Im5; (iii) colicin D with Im; (iv) E7-Im7; (v) E9-Im9. While the PPIs of the first 3 types were recognized to be distinct and to belong to different classes, the interactions of E7-Im7 and E9-Im9 were observed to be extremely similar. Specifically, we have aligned 6 PPIs of E9-Im9 (1bxiAB, 1emvAB, 1fr2AB) and E7-Im7(1mz8AB, 1ujzAB, 1fr2AB) and observed 7 conserved interactions (see  in Additional file 2). Four shared interactions (two hydrogen bonds and two aromatic interactions) are created by the conserved YY motif (Tyr-54, Tyr-55, 1bxi numbering) [60]. The rest of the conserved interactions are the hydrogen bonds formed by Glu-30 and Asp-51 and a hydrophobic aliphatic interaction formed by Pro-56. The results of MAPPIS are consistent with alanine scanning, and residues Asp-51, Tyr-55 and Pro-56 are indeed hotspots with ΔΔ*G *of 5.9, 4.6 and 1.24 *kcal/mol *respectively. In addition, the results of MAPPIS are consistent with previous biological studies [60], which emphasized the conservation of the interaction of Tyr-55 (E9) with Phe-86 (Im9). Interestingly, we observed that, due to reduction of the number of false positives, interactions shared between E9-Im9 and E7-Im7 provided a better prediction of hot spots than interactions shared only by the PPIs of E9-Im9. Specifically, the success rate of the predictions based on the three PPIs of E9-Im9 is only 0.58 (ROC area 0.47, specificity 0.38 and sensitivity 0.78). Taking into consideration the additional three complexes of E7-Im7 increases the specificity of the predictions and achieves the success rate of 0.72 (ROC area 0.72, specificity 1.0 and sensitivity 0.44. As can be seen in Figure 2B MAPPIS maximizes the similarity in the interface area and allows to overcome the backbone flexibility and the rotation of the overall structures. These were described by Joachimiak et al. [61], who have designed a new interface of E7-Im7. Interestingly, when we added the structure of the redesigned PPI (2erh) to our alignment, the shared pattern of interactions, detailed in Figure 2B remained almost unchanged. Most of the amino acids that create it were not modified and even those that were mutated preserved the interaction. For example, one of the interactions conserved in all the PPIs was created by the backbone *O *of Gln-528 in E7 (1mz8). Using MAPPIS, we observed that a similar backbone interaction was present in the redesigned PPI (2erh), in which this amino-acid was mutated to Lys-528. This example shows that MAPPIS can be used to guide protein design studies. It can recognize the most crucial interactions, which should remain unchanged and can point to amino acids that are not crucial for interaction or interact via their backbone atoms and can be replaced.

### PPIs of superantigens with T-Cell receptors

Superantigens (SAGs) are a group of toxins that activate T-cells causing system-wide inflammation and other human diseases. Sundberg et al. [62] have analyzed complexes of different SAGs with T cell receptors (TCRs) and observed a diversity of binding modes backbone conformations. Intrigued by this phenomenon, we applied MAPPIS to align 6 complexes of TCRs with SAGs: (1) SEC3 (PDB: 1jckAB, 2aq3AB); (2) SEB (PDB:1sbbAB); (3) SpeA (PDB:1l0yAB, 1l0xAB) and (4) SpeC (PDB:1ktkEA). Remarkably, within 1 second, which is the running time of MAPPIS, we obtained results that are consistent with the thorough manual analysis of the interactions in each type of complexes done by Sundberg et al. [62]. Although the overall backbones of the compared SAGs can not be rigidly aligned in 3D space (see figure 2 in [62]), the chemical binding organizations of their complexes are similar. Specifically, we recognized 4 spatially conserved interactions (1 aromatic and 3 hydrogen bonds, see Table 4 in Additional file 2). Notably, all of these shared interactions are created by experimentally verified hot spots. Moreover, two of these interactions are created by pairs of cooperative hot spots that interact across the interface: Gly-53 and Thr-55 of TCR that interact with the hot spots Gln-210 and Asn-23 of SAG respectively (1jck numbering). Most of the shared interactions, which are spread along the TCR regions CDR2/FR3, were detailed by Sundberg et al. for each of the complexes with SpeA, SpeC and SEB (see figure 4 in [62]). Although the compared complexes have different binding conformations, MAPPIS aligns the loops in the binding regions and overcomes the backbone flexibility. Moreover, as these spatially conserved interactions are created by amino acids with different identities, the similarities described above can not be recognized by residue based computational methods.

## Conclusion

Here, we have shown that spatially conserved physico-chemical interactions play a crucial functional role. We have presented a computational method, MAPPIS, for recognition of such patterns of conserved interactions formed between groups of atoms independent of the identity of the amino acids as well as the overall protein sequences and folds. Considering multiple complexes of functionally similar PPIs, MAPPIS allows the identification of the smallest set of interactions that may be responsible for binding and function. We have shown that chemical groups that form spatially conserved interactions correlate with cooperative effects in double mutant cycles and are useful for predicting hot spots.

Interestingly, we observed that increasing the number of the compared PPIs, as well as comparing PPIs of proteins with different overall sequences and folds, improves the specificity of the hot spot prediction. The main limitation of our approach is the requirement for the existence of a sufficient number of high resolution structures of complexes comprised by functionally similar proteins. The selection of such complexes is not straightforward, especially as there is no direct correspondence between functional similarity and the similarity of the overall sequences and structures [32].

With the fast progress of Structural Genomics and the availability of multiple structures of functionally related proteins, methods like MAPPIS are expected to become increasingly useful. MAPPIS complements both computational and experimental alanine scanning by explaining the functional role of hot spots in the formation of atomic interactions. Further, by recognition of conserved spatial patterns of physico-chemical interactions, it rationalizes hot spots' cooperativity and elucidates the complex binding organizations of the protein-protein interfaces. Therefore, it complements the experimental techniques, such as the double mutant cycle, which provide the experimental evidence for the cooperativity effects at the amino acid level but do not describe the atomic interactions that are responsible for it. Moreover, analysis of the conserved interactions with MAPPIS can explain the effect of amino acids' mutations and can contribute to studies of the binding affinity and specificity. Furthermore, targeting the conserved chemical organizations may be a useful strategy in protein and drug design.

## The MAPPIS Method

**Figure 3:**
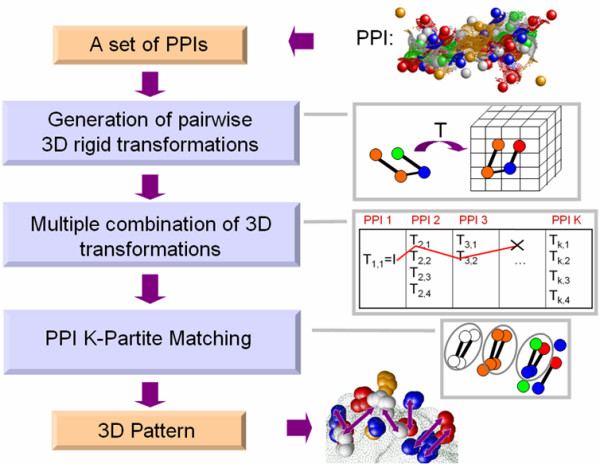
Overview of the MAPPIS method.

Given a set of PPIs, MAPPIS solves an optimization problem of finding a set of transformations that superimpose the PPIs in 3D space in a way that maximizes the spatial and chemical similarity of their interactions and pseudocenters. As this optimization problem is computationally NP-hard [58], we provide an efficient approximation algorithm, the main stages of which are presented in Figure 3 and below.

### The input

The input to MAPPIS consists of *K *PPIs {Ii=(Ai,Bi)}i=1K
 MathType@MTEF@5@5@+=feaafiart1ev1aaatCvAUfKttLearuWrP9MDH5MBPbIqV92AaeXatLxBI9gBaebbnrfifHhDYfgasaacH8akY=wiFfYdH8Gipec8Eeeu0xXdbba9frFj0=OqFfea0dXdd9vqai=hGuQ8kuc9pgc9s8qqaq=dirpe0xb9q8qiLsFr0=vr0=vr0dc8meaabaqaciaacaGaaeqabaqabeGadaaakeaacqGG7bWEcqWGjbqsdaWgaaWcbaGaemyAaKgabeaakiabg2da9iabcIcaOiabdgeabnaaBaaaleaacqWGPbqAaeqaaOGaeiilaWIaemOqai0aaSbaaSqaaiabdMgaPbqabaGccqGGPaqkcqGG9bqFdaqhaaWcbaGaemyAaKMaeyypa0JaeGymaedabaGaem4saSeaaaaa@3FC7@. These are represented by their physico-chemical properties and interactions as presented in Figure 1A (see Results). For *K *interfaces we define the similarity with respect to the *pivot *PPI, which is selected as the first PPI. We assume that we are given the correspondence between the compared protein chains (i.e. *A*_*i *_corresponds to *B*_*i*_). This correspondence can be obtained either from the biological data (e.g. molecule names) or by running the pairwise alignment between (*A*_1_, *B*_1_) - (*A*_*i*_, *B*_*i*_) and (*A*_1_, *B*_1_) - (*B*_*i*_, *A*_*i*_), for each *i *≠ 1.

### Generation of pairwise transformations

Given a set of PPIs, we create a set of pairwise transformations that can superimpose each PPI the pivot. These transformations are constructed based on the information of the physico-chemical interactions formed across each interface. Specifically, each pair of pivot interactions is stored in a four-dimensional hash table with a key encoding the interactions' lengths and the distances between pseudocenters as well as their physico-chemical properties. Each pair of interactions from each PPI except the pivot is used to access the hash table and retrieve similar interaction pairs of the pivot. Each pair of matched interactions defines a candidate transformation that can superimpose the considered PPI upon the pivot. In particular, we use the least square fitting method and given two interactions from two PPIs, (aij,bij)∈Ij
 MathType@MTEF@5@5@+=feaafiart1ev1aaatCvAUfKttLearuWrP9MDH5MBPbIqV92AaeXatLxBI9gBaebbnrfifHhDYfgasaacH8akY=wiFfYdH8Gipec8Eeeu0xXdbba9frFj0=OqFfea0dXdd9vqai=hGuQ8kuc9pgc9s8qqaq=dirpe0xb9q8qiLsFr0=vr0=vr0dc8meaabaqaciaacaGaaeqabaqabeGadaaakeaacqGGOaakcqWGHbqydaqhaaWcbaGaemyAaKgabaGaemOAaOgaaOGaeiilaWIaemOyai2aa0baaSqaaiabdMgaPbqaaiabdQgaQbaakiabcMcaPiabgIGiolabdMeajnaaBaaaleaacqWGQbGAaeqaaaaa@3BDC@ and (ait,bit)∈It
 MathType@MTEF@5@5@+=feaafiart1ev1aaatCvAUfKttLearuWrP9MDH5MBPbIqV92AaeXatLxBI9gBaebbnrfifHhDYfgasaacH8akY=wiFfYdH8Gipec8Eeeu0xXdbba9frFj0=OqFfea0dXdd9vqai=hGuQ8kuc9pgc9s8qqaq=dirpe0xb9q8qiLsFr0=vr0=vr0dc8meaabaqaciaacaGaaeqabaqabeGadaaakeaacqGGOaakcqWGHbqydaqhaaWcbaGaemyAaKgabaGaemiDaqhaaOGaeiilaWIaemOyai2aa0baaSqaaiabdMgaPbqaaiabdsha0baakiabcMcaPiabgIGiolabdMeajnaaBaaaleaacqWG0baDaeqaaaaa@3C18@, *i *= 1, 2, we compute a transformation that can best superimpose them in 3D space, i.e. a transformation that minimizes the RMSD between the pseudocenters: (∑i|aij−T∗(ait)|2+∑i|bij−T∗(bit)|2)14
 MathType@MTEF@5@5@+=feaafiart1ev1aaatCvAUfKttLearuWrP9MDH5MBPbIqV92AaeXatLxBI9gBaebbnrfifHhDYfgasaacH8akY=wiFfYdH8Gipec8Eeeu0xXdbba9frFj0=OqFfea0dXdd9vqai=hGuQ8kuc9pgc9s8qqaq=dirpe0xb9q8qiLsFr0=vr0=vr0dc8meaabaqaciaacaGaaeqabaqabeGadaaakeaadaGcaaqaaiabcIcaOmaaqababaGaeiiFaWNaemyyae2aa0baaSqaaiabdMgaPbqaaiabdQgaQbaakiabgkHiTiabdsfaunaaCaaaleqabaGaey4fIOcaaOGaeiikaGIaemyyae2aa0baaSqaaiabdMgaPbqaaiabdsha0baakiabcMcaPiabcYha8naaCaaaleqabaGaeGOmaidaaOGaey4kaSYaaabeaeaacqGG8baFcqWGIbGydaqhaaWcbaGaemyAaKgabaGaemOAaOgaaOGaeyOeI0Iaemivaq1aaWbaaSqabeaacqGHxiIkaaGccqGGOaakcqWGIbGydaqhaaWcbaGaemyAaKgabaGaemiDaqhaaOGaeiykaKIaeiiFaW3aaWbaaSqabeaacqaIYaGmaaGccqGGPaqkdaWcaaqaaiabigdaXaqaaiabisda0aaaaSqaaiabdMgaPbqab0GaeyyeIuoaaSqaaiabdMgaPbqab0GaeyyeIuoaaSqabaaaaa@5B1F@. As we construct only the transformations that can superimpose at least two physico-chemically similar interactions, we reduce the number of the constructed transformations and achieve a performance gain over other methods (e.g. MultiBind, see Table 1 in Additional file 2).

### Multiple combination of 3D transformations

At the next stage we construct the multiple alignments which are based on the combination of all the candidate pairwise transformations constructed at the previous stage. The number of possible combinations is exponential in the number of PPIs. To practically overcome this limitation we apply an efficient *branch-and-bound *technique that effectively filters out a large number of low scoring solutions [58]. As illustrated in Figure 3, we iteratively traverse the created transformations. Each time we create a multiple alignment of a set of *m *PPIs and try to add a transformation Tim+1
 MathType@MTEF@5@5@+=feaafiart1ev1aaatCvAUfKttLearuWrP9MDH5MBPbIqV92AaeXatLxBI9gBaebbnrfifHhDYfgasaacH8akY=wiFfYdH8Gipec8Eeeu0xXdbba9frFj0=OqFfea0dXdd9vqai=hGuQ8kuc9pgc9s8qqaq=dirpe0xb9q8qiLsFr0=vr0=vr0dc8meaabaqaciaacaGaaeqabaqabeGadaaakeaacqWGubavdaqhaaWcbaGaemyAaKgabaGaemyBa0Maey4kaSIaeGymaedaaaaa@329A@ of the PPI, *I*_*m*+1_. However, if an estimated score of the multiple alignment between these *m *+ 1 PPIs is lower than the score of the best multiple alignment found so far between all the *K *input PPIs (*K *≤ *m*), we can ignore this combination of transformations and there is no need to try to extend it. Essentially, we continue and try to add another transformation, Ti+1m+1
 MathType@MTEF@5@5@+=feaafiart1ev1aaatCvAUfKttLearuWrP9MDH5MBPbIqV92AaeXatLxBI9gBaebbnrfifHhDYfgasaacH8akY=wiFfYdH8Gipec8Eeeu0xXdbba9frFj0=OqFfea0dXdd9vqai=hGuQ8kuc9pgc9s8qqaq=dirpe0xb9q8qiLsFr0=vr0=vr0dc8meaabaqaciaacaGaaeqabaqabeGadaaakeaacqWGubavdaqhaaWcbaGaemyAaKMaey4kaSIaeGymaedabaGaemyBa0Maey4kaSIaeGymaedaaaaa@346C@ of *I*_*m*+1_, and so on. Although theoretically the number of such traversals may be exponential, the filtering is very efficient and leads to low running times.

Furthermore, we achieve an additional speed up by the observation that we do not need to actually construct a multiple alignment for each set of *m *+ 1 PPIs, but we can estimate an upper bound on its score. In particular, we calculate the highest score that can be achieved between the superimposed pseudocenters, without the requirement for the exact correspondence which resolves multiple matches.

### Construction of the common pattern

For each potentially high scoring multiple superposition we compute the exact correspondence between the superimposed pseudocenters and interactions and determine the common pattern. The calculation of such correspondence involves solving a problem of PPI K-partite matching which is NP-hard even for a pair of PPIs [50]. Here, we implement the following greedy algorithm. First, we sort the superimposed interactions and pseudocenters according to their physico-chemical score (see Additional file 3). Each time, we greedily select a highest scoring set of multiply matched interactions (one from each PPI) and mark the selected pseudocenters as matched. The next selection will be made from the still unmatched pseudocenters. Where the number of interactions in which each pseudocenter can participate is bounded by the valency of the atoms. Once we have determined the pattern of interactions we apply a similar greedy procedure to determine the set of matched non-interacting pseudocenters. All candidate patterns are scored by the physico-chemical scoring functions which is detailed in Additional file 3. In all of the described examples (see Section Results) we have referred only to a single solution which received the highest score.

#### Running Time Complexity

The time complexity depends mainly on the stage of the multiple combination of 3D transformations and it is bounded by *O*(*n*^3*K'*^*nK *log(*n*)), where *n *is the number of pseudocenters in the largest PPI and *K' *is the depth of branch-and-bound stage (*K' *≤ *K*) [50]. The practical running times of MAPPIS are as low as reported in Table 1 in Additional file 2

## Availability and requirements

The MAPPIS software is available for download at: . The software package contains the executable and a set of Perl scripts for PPI extraction. The package is suitable for the Linux platform and its download is free for non-commercial users.

## Competing interests

The author(s) declares that there are no competing interests.

## Authors' contributions

AS-P and MS developed the MAPPIS method. All authors participated in the research design and manuscript preparation.

## Supplementary Material

Additional file 1 Supplementary figures. Click here for file

Additional file 2 Supplementary tables. Click here for file

Additional file 3 The Physico-Chemical Scoring Function. Click here for file
