# Supplementary material for: Spatial chemical conservation of hot spot interactions in protein-protein complexes
Source: BMC Biol. 2007 Oct 9;5:43. doi: 10.1186/1741-7007-5-43 (PMC2231411; doi:10.1186/1741-7007-5-43)
Supplement: Additional file 1 — Supplementary figures. [file 1741-7007-5-43-S1.pdf]

Additional File 1: Supplementary Figures

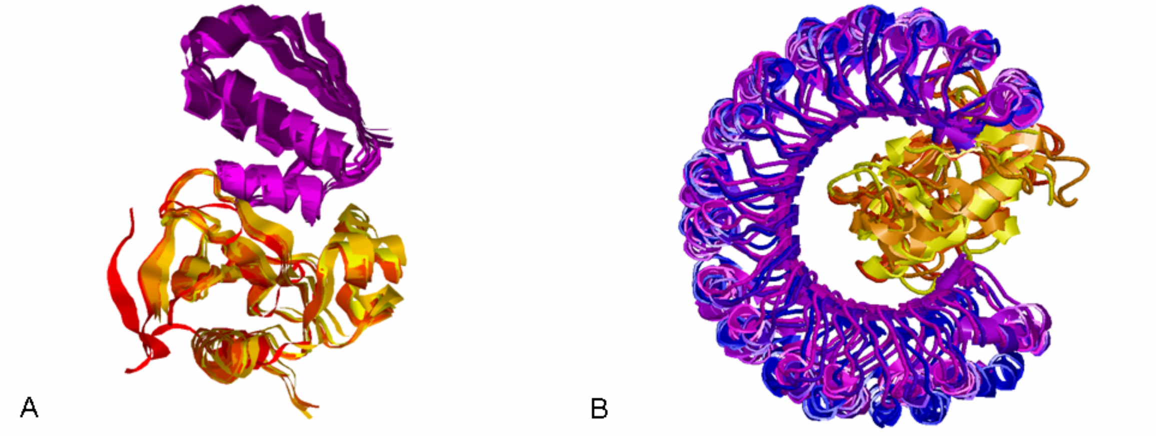

Figure 1: (**A**) Superimposition of the 6 barnase-barstar complexes obtained by the alignment of MAPPIS. The PDB codes are: 1brsAD,1b2sAD,1b27AD,1x1uAD,1x1wAD,1b2uAD. The structures of barnase are colored ranging from yellow (1brsAD) to red (1ay7AB) respectively. The structures of barstar range from magenta (1brsAD) to purple (1b2uAD) in the same order. (**B**) Alignment of 4 complexes of Ribonucleases with inhibitors (PDB codes:1a4yAB, 2bexAC, 1z7xZY, 1dfjIE). The colors of Ribonucleases range from yellow (1a4yB to red 1dfjE). The colors of the inhibitors range from light purple (1a4yA) to dark blue (1dfjI). As can be seen MAPPIS aligns the interactions in the interface area and overcomes the flexibility of the C-terminal end of the RIs and the loops of ribonucleases. The details of the shared interactions are provided in Table 3 in Additional file 1.

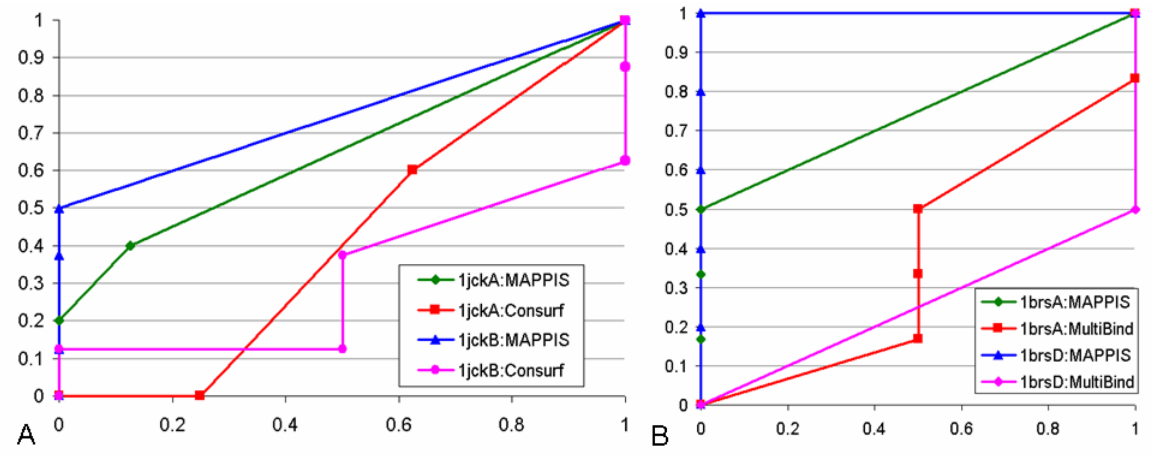

Figure 2: (**A**) The ROC curves of MAPPIS and Consurf constructed for the prediction of hot spots of PPIs of superantigens with T-Cell receptors (PDB: 1jck). The curves plot the sensitivity (the true positive rate, $TP/(TP + FN)$) as the function of the true negative rate (1-specificity, $TN/(TN + FP)$), while varying the prediction threshold. The curves of MAPPIS are constructed by varying the threshold for the similarity of physico-chemical interactions, where similar interactions with a score lower than the threshold are not used for the prediction of hot spots. The curves of Consurf are constructed by varying the conservation score, which is used as a threshold for hot spots prediction. The graphs that illustrate the quality of the predictions made for the T cell receptor (1jckA) by MAPPIS and Consurf are depicted in green and red respectively. The curves that plot the prediction of the superantigen (1jckB) hot spots by MAPPIS and Consurf are blue and pink. (**B**) The ROC curves of MAPPIS and MultiBind constructed for the prediction of hot spots in the PPI of barnase-barstar (1brs). The MAPPIS threshold is varied as in (A). The curves of MultiBind are constructed by varying the threshold for the similarity of physico-chemical properties of the binding sites, where similar pseudocenters with a score lower than the threshold are not used for the prediction of hot spots. The graphs that illustrate the quality of the predictions made by MAPPIS and MultiBind for the barnase (1brsA) are depicted in green and red respectively. The curves that plot the prediction of the barstar (1brsD) hot spots by MAPPIS and MultiBind are blue and pink.
