# Supplementary material for: Spatial chemical conservation of hot spot interactions in protein-protein complexes
Source: BMC Biol. 2007 Oct 9;5:43. doi: 10.1186/1741-7007-5-43 (PMC2231411; doi:10.1186/1741-7007-5-43)
Supplement: Additional file 2 — Supplementary tables. [file 1741-7007-5-43-S2.pdf]

Additional File 2: Supplementary Tables

PDB: Chain	Mol. num.	**Mappis**					**MultiBind**				**MultiProt**		PPI size
		ROC area	Spec.	Sens.	Run time (min)	TP,TN, FP,FN	ROC area	Spec.	Sens.	Run time (min)	Spec.	Sens.	
1a4y:A	4	0.74	0.78	0.75	0.4	3,7,2,1	0.5	0	1	94	0	1	220
1brs:A	6	0.75	1	0.5	9	3,2,0,3	0.38	0	1	94	0	1	133
1brs:D	6	1	1	0.8	9	5,1,0,0	0.58	1	0.17	63	0	1	133
1cbw:I	7	1	0.88	1	5	1,6,2,0	0.6	0.81	0.33	45.5	0	1	112
1gc1:C	6	0.74	0.86	0.67	1.2	2,18,3,1	0.6	0.81	0.33	45.5	0	1	184
1bxi:A	6	0.67	1	0.44	0.5	4,8,0,5	0.73	0.75	0.78	30.3	0.12	0.78	108
1dan:L	6	1	0.73	1	2.1	3,8,3,0	0.7	0.18	1	61	1	0	279
1jck:A	6	0.65	0.88	0.4	1	2,7,1,3	0.59	0.38	0.8	36.5	0.38	0.2	114
1jck:B	6	0.75	1	0.38	1	4,2,0,4	0.5	1	0	36.5	1	0.5	114
1vfb:C	7	0.51	0.62	0.5	68	1,7,2,2	0.5	0	1	189	0	1	121
3hfm:Y	8	0.62	0.6	0.67	39	2,6,4,1	0.6	0.1	1	720	0	1	141
3hhr:A	4	0.74	1	0.52	0.5	13,16,0,12	0.64	0.31	0.88	39	1	0	198
	Mean Mappis						Mean MultiBind				Mean MultiProt		
	6	0.76	0.86	0.64	11.4		0.58	0.44	0.69	121.2	0.29	0.7	154

Table 1: **Comparison of MAPPIS to MultiProt [1] and MultiBind [2]**

1a4y:RI(A)-Angiogenin(B)			2bex:RI(A)-neurotoxin(C)			1z7x:RI(Z)-RNaseI(Y)			1dfj:RI(I)-RnaseA(E)			
Chain. R. Num	R. Type	Psc. Type	Chain R. Num	R. Type	Psc. Type	Chain R. Num	R. Type	Psc. Type	Chain R. Num	R. Type	Psc. Type	Type
A.432	Val	ALI	A.432	Val	ALI	Y.432	Val	ALI	I.428	Val	ALI	s
B.38	Pro	ALI	C.36	Arg	ALI	Z.39	Arg	ALI	E.39	Arg	ALI	s
A.434	Tyr	DAC	A.434	Tyr	DAC	Y.434	Tyr	DAC	I.430	Tyr	DAC	s
B.38	Pro	ACC	C.36	Arg	ACC	Z.39	Arg	ACC	E.39	Arg	ACC	b
A.435	Asp	PI:C	A.435	Asp	PI:C	Y.435	D	PI:C	I.431	Asp	PI:C	b
B.114	His	PI	C.129	His	PI	Z.119	His	PI	E.119	His	PI	s
A.436	Ile	PI:C	A.436	Ile	PI:C	Y.436	Ile	PI:C	I.432	Thr	PI:C	b
B.114	His	PI	C.129	His	PI	Z.119	His	PI	E.119	His	PI	s
A.437	Tyr	PI	A.437	Tyr	PI	Y.437	Tyr	PI	I.433	Tyr	PI	s
B.114	His	PI	C.129	His	PI	Z.119	His	PI	E.119	His	PI	s
A.459	Ile	ALI	A.459	Ile	ALI	Y.459	Ile	ALI	I.455	Ile	ALI	s
B.38	Pro	ALI	C.36	Arg	ALI	Z.39	Arg	ALI	E.39	Arg	ALI	s
A.460	Ser	DAC	A.460	Ser	DAC	Y.460	Ser	ACC	I.456	Ser	DAC	
B.8	His	DAC	C.10	Trp	DON	Z.7	Lys	DON	E.7	Lys	DON	s

Table 2: **The interactions shared by 4 PPis of RNase A-like ribonucleases with leucine-rich repeat inhibitors**. Each pair of rows details the interacting pseudocenters of two PPI chains. Each three columns present the details of a specific PPI: (i) chain identifier and residue number; (ii) residue type; (iii) pseudocenter type, which can be donor (DON), acceptor (ACC), mixed donor/acceptor (DAC), hydrophobic aliphatic (ALI) or aromatic (PI). The last column presents the origin of the feature: backbone(b) or side-chain(s) if it is the same for all the matched pseudocenters.

PPI 1: 1bxiAB			PPI 2: 1fr2AB			PPI 3: 1emvAB			PPI 4: 1mz8AB			PPI 5: 1znvAB			PPI 6: 1ujzAB			
Chain. R. Num	R. Type	Psc. Type	Chain R. Num	R. Type	Psc. Type	Chain R. Num	R. Type	Psc. Type	Chain R. Num	R. Type	Psc. Type	Chain R. Num	R. Type	Psc. Type	Chain R. Num	R. Type	Psc. Type	Type
A.30	Glu	ACC	A.30	Glu	ACC	A.30	Glu	ACC	A.31	Asp	ACC	A.31	Asp	ACC	A.31	Asp	ACC	s
B.84	Ser	DON	B.84	Ser	DON	B.84	Ser	DON	B.520	Arg	DON	B.520	Arg	DON	B.520	Arg	DON	
A.51	Asp	ACC	A.51	Asp	ACC	A.51	Asp	ACC	A.52	Asp	ACC	A.52	Asp	ACC	A.52	Asp	ACC	s
B.89	Lys	DON	B.89	Lys	DON	B.89	Lys	DON	B.531	Thr	DON	B.531	Thr	DON	B.531	Thr	DON	b
A.54	Tyr	ACC	A.54	Tyr	ACC	A.54	Tyr	ACC	A.55	Tyr	ACC	A.55	Tyr	ACC	A.55	Tyr	ACC	b
B.72	Asn	DON	B.72	Asn	DON	B.72	Asn	DON	B.514	Ser	DAC	B.514	Ser	DAC	B.514	Ser	DAC	s
A.54	Tyr	PI	A.54	Tyr	PI	A.54	Tyr	PI	A.55	Tyr	PI	A.55	Tyr	PI	A.55	Tyr	PI	s
B.87	Thr	PI:C	B.87	Thr	PI:C	B.87	Thr	PI:C	B.529	Thr	PI:C	B.529	Thr	PI:C	B.529	Thr	PI:C	b
A.55	Tyr	DAC	A.55	Tyr	DAC	A.55	Tyr	DAC	A.56	Tyr	DAC	A.56	Tyr	DAC	A.56	Tyr	DAC	s
B.86	Phe	ACC	B.86	Phe	ACC	B.86	Phe	ACC	B.528	Lys	ACC	B.528	Lys	ACC	B.528	Lys	ACC	b
A.55	Tyr	PI	A.55	Tyr	PI	A.55	Tyr	PI	A.56	Tyr	PI	A.56	Tyr	PI	A.56	Tyr	PI	s
B.99	Tyr	PI	B.99	Tyr	PI	B.99	Tyr	PI	B.541	Phe	PI	B.541	Phe	PI	B.541	Phe	PI	s
A.56	Pro	ALI	A.56	Pro	ALI	A.56	Pro	ALI	A.57	Pro	ALI	A.57	Pro	ALI	A.57	Pro	ALI	s
B.73	Pro	ALI	B.73	Pro	ALI	B.73	Pro	ALI	B.515	Arg	ALI	B.515	Arg	ALI	B.515	Arg	ALI	s

Table 3: **The interactions shared by 6 PPIs of colicins with immunity proteins** The interactions recognized by MAPPIS to be shared between 6 PPIs of E9-Im9 (1bxiAB, 1emvAB, 1fr2AB) and E7-Im7(1mz8AB, 1ujzAB, 1fr2AB). The columns headers are as in Table 2.

PPI 1: 1jckAB			PPI 2: 2aq3AB			PPI 3: 1sbbAB			PPI 4: 1l0yAB			PPI 5: 1l0xAB			PPI 6: 1ktkEA			
Chain. R. Num	R. Type	Psc. Type	Chain R. Num	R. Type	Psc. Type	Chain R. Num	R. Type	Psc. Type	Chain R. Num	R. Type	Psc. Type	Chain R. Num	R. Type	Psc. Type	Chain R. Num	R. Type	Psc. Type	Type
A.52	Ala	PI:C	A.53	Gly	PI:C	A.52	Ala	PI:C	A.52	Ala	PI:C	A.52	Ala	PI:C	E.51	Glu	PI:C	b
B.90	Tyr	PI	B.90	Tyr	PI	B.90	Tyr	PI	B.84	Tyr	PI	B.84	Tyr	PI	A.78	Leu	PI	
A.53	Gly	ACC	A.53	Gly	ACC	A.53	Gly	ACC	A.53	Gly	ACC	A.53	Gly	ACC	E.52	Ser	DAC	
B.210	Gln	DON	B.208	Gln	DON	B.210	Gln	DON	B.194	Gln	DON	B.194	Gln	DON	A.181	Arg	DON	s
A.55	Thr	DON	A.55	Thr	DON	A.55	Thr	DON	A.55	Thr	DON	A.55	Thr	DON	E.69	His	DON	b
B.23	Asn	ACC	B.23	Asn	ACC	B.23	Asn	ACC	B.20	Asn	ACC	B.20	Asn	ACC	A.15	Tyr	DAC	s
A.70	Pro	ACC	A.70	Pro	ACC	A.70	Pro	ACC	A.70	Pro	ACC	A.70	Pro	ACC	E.28	Gln	ACC	b
B.60	Asn	DON	B.60	Asn	DON	B.60	Asn	DON	B.54	Asn	DON	B.54	Asn	DON	A.79	Asn	DON	s

Table 4: **The interactions shared by 6 PPIs of T-cell receptors with superantigens**. The interactions recognized by MAPPIS to be shared between 6 PPIs of TCRs with SAGs: (1) SEC3 (PDB: 1jckAB, 2aq3AB); (2) SEB (PDB:1sbbAB); (3) SpeA (PDB:1l0yAB, 1l0xAB) and (4) SpeC (PDB:1ktkEA). The columns headers are as in Table 2.

References

1. Shatsky M, Nussinov R, Wolfson HJ: **A method for simultaneous alignment of multiple protein structures**. *Proteins* 2004, **56**:143–156.
2. Shatsky M, Shulman-Peleg A, Nussinov R, Wolfson H: **The multiple common point set problem and its application to molecule binding pattern detection**. *J Comput Biol.* 2006, **13**:407–42.
