# Supplementary material for: Spatial chemical conservation of hot spot interactions in protein-protein complexes
Source: BMC Biol. 2007 Oct 9;5:43. doi: 10.1186/1741-7007-5-43 (PMC2231411; doi:10.1186/1741-7007-5-43)
Supplement: Additional file 3 — The Physico-Chemical Scoring Function. [file 1741-7007-5-43-S3.pdf]

Additional File 3: The Physico-Chemical Scoring Function

Similarity between two superimposed interactions $i = (a, b)$ and $i' = (a', b')$ is measured by [1]:

$$S(i, i') = S_{IN}(i) + S_{IN}(i') + S_{PC}(a, a') + S_{PC}(b, b')$$

$$S_{IN}(i) = propen(i) \cdot \begin{cases} 0, & dist(i) > max\_dist(i) \\ (max\_dist(i) - dist(i))/(1 + charge\_comp(i)) & chem(i) = HB \\ (max\_dist(i) - dist(i))/(1 + shape\_comp(i)) & chem(i) = ALI \\ (max\_dist(i) - dist(i))/(1 + shape\_comp(i) + n_{PI}(i)) & chem(i) = PI \end{cases}$$

The similarity between two superimposed pseudocenters is defined by [2]:

$$S_{PC}(a, b) = \begin{cases} 0, & dist(a,b) > max\_dist(a,b) \text{ or } chem(a) \neq chem(b) \\ 0, & shape(a,b) > 0.2 \text{ or } n_S(a,b) > 0.2 \\ (max\_dist(a,b) - dist(a,b))/(1 + charge(a,b)) & chem(a) = HB \\ (max\_dist(a,b) - dist(a,b))/(1 + shape(a,b) + n_{PI}(a,b)) & chem(a) = PI \\ (max\_dist(a,b) - dist(a,b) + v_{ALI}(a,b))/(2 + 20 * shape(a,b)) & chem(a) = ALI \end{cases}$$

- $dist(a, b)$ - the distance between a and b after the superimposition. $dist(i)$ - the distance between interacting pseudocenters a and b.
- $chem(a)$, $chem(i)$ - the physico-chemical property of the point a or interaction i. There are three types of properties: Hydrogen Bonding (HB), Aliphatic Hydrophobic (ALI) and Aromatic (PI).
- $max\_dist(a, b)$ - maximal allowed distance between a pair of pseudocenters, defined by ε =3.0Å.

$max\_dist(i)$ - the maximal distance allowed for the specific type of interaction. The default thresholds are γ =3.9Å for hydrogen bonds [3] and γ =8.0Å for hydrophobic aliphatic and aromatic interactions.

bridges.

- $charge(a)$ - the partial atomic charge of the atom a, which can form hydrogen bonds.

$charge(a, b) = |charge(a) - charge(b)|$ - measures the similarity of charges.

$charge\_comp(i) = |charge(a) + charge(b)|$ - measures the complementarity of charges.

- $shape(a)$ - the average curvature of the surface region created by a. Calculated as an average of the solid angle shape functions [4] with spheres of radius 4,5,6 and 7Å . The sphere centers are located at projection point of a to the surface. $shape(a, b) = |shape(a) - shape(b)|$ - measures the similarity of shapes.

$shape\_comp(i) = |1 - shape(a) - shape(b)|$ - measures the complementarity of shapes which sums to one.

- $n_S(a)$ - normal vector at the projection point of a to the surface, $n_S(a, b) = n_S(a) \cdot n_S(b)$.
- $v_{ALI}(a, b)$ - the overlap of the hydrophobic group bounding spheres of a and b, approximated by the difference between sum of radiuses and the distance between the centers.
- $n_{PI}(a)$ - for aromatic pseudocenters denotes the normal to the plane of the aromatic ring.

$n_{PI}(i) = n_{PI}(a) \cdot n_{PI}(b)$ - represents the angle between two interacting aromatic ring.

• $propen(p)$ - the propensity of the physico-chemical property in the interface compared to the overall protein chain. The propensities of the pseudocenters were calculated by Mintz et al. [5].

$propen(i) = propen(a) \cdot propen(b)$.

References

1. Shulman-Peleg A, Shatsky M, Nussinov R, Wolfson H: **MAPPIS: Multiple 3D Alignment of Protein-Protein Interfaces**. In *Complife, Konstanz, Germany, Springer Lec. Notes in Comp. Sci.*, *Volume 3695*. Edited by Berthold M 2005:91–103.
2. Shatsky M, Shulman-Peleg A, Nussinov R, Wolfson H: **Recognition of Binding Patterns Common to a Set of Protein Structures**. In *RECOMB 2005, Cambridge MA*, *Volume 3500*. Edited by Miyano S, LNCS 2005:440–455.
3. McDonald IK, Thornton JM: **Satisfying hydrogen bonding potential in proteins**. *J. Mol. Biol.* 1994, **238**:777–793.
4. Connolly ML: **Measurement of protein surfaces shape by solid angles**. *J. Mol. Graph.* 1986, **4**:3–6.
5. Mintz S, Shulman-Peleg A, Wolfson HJ, Nussinov R: **Generation and analysis of a protein-protein interface dataset with similar chemical and spatial patterns of interactions**. *Proteins* 2005, **61**:6–20.
